# Supplementary figures and images for: Hookworm genomic diversity and population structure from accessible sample types: A validated approach to generate genome-wide polymorphism datasets from individual third-stage larvae
Source: bioRxiv. 2026 Feb 20:2026.01.06.697970. Originally published 2026 Jan 7. Preprint. [Version 2] doi: 10.64898/2026.01.06.697970 (PMC12803160; doi:10.64898/2026.01.06.697970)

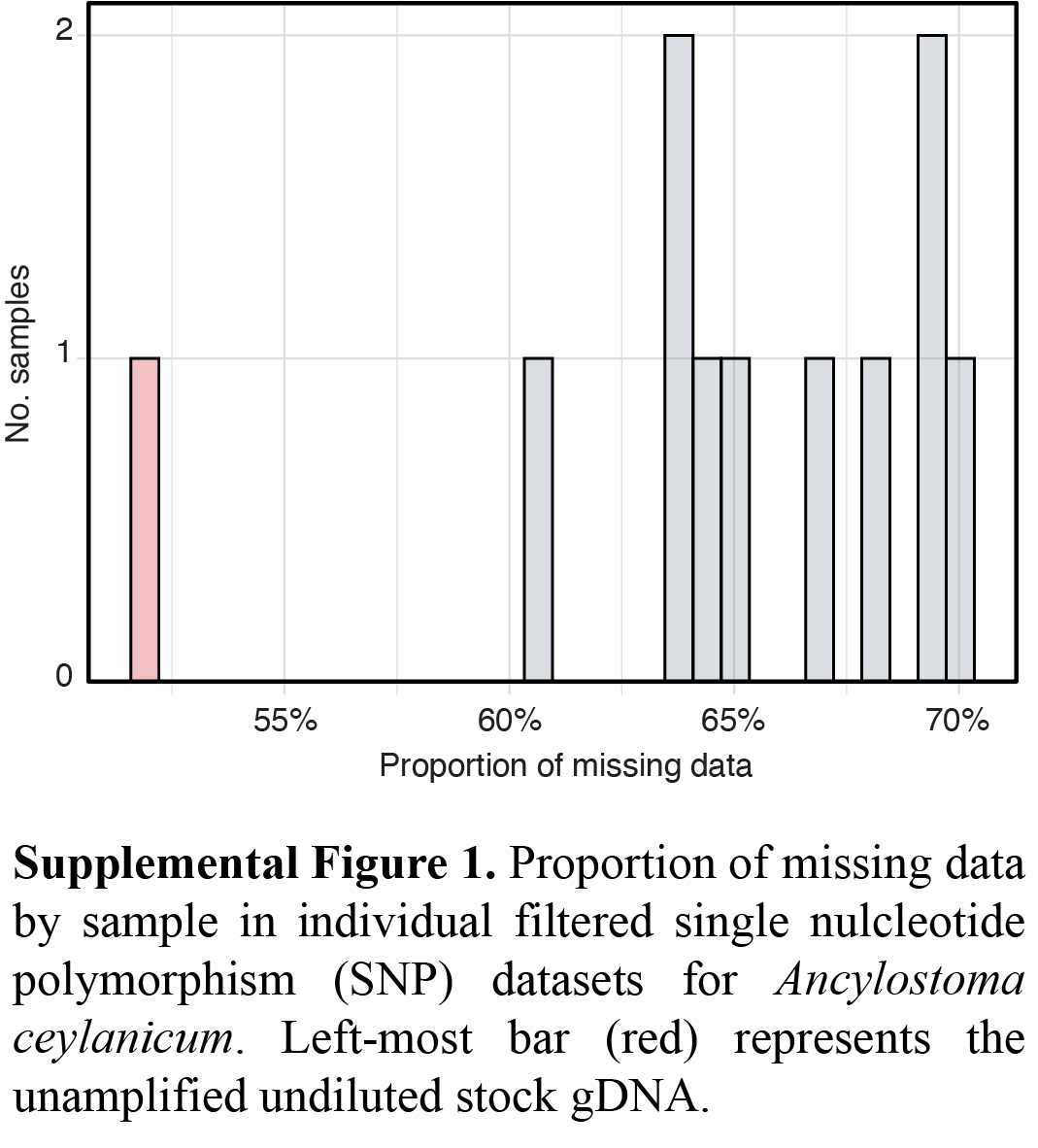

Supplement: Supplement 1 [file media-1.jpg]
